# Crystallization Behavior of the Low-Temperature Mineralization Sintering Process for Glass Nanoparticles

**DOI:** 10.3390/ma13153281

**Published:** 2020-07-23

**Authors:** Yeongjun Seo, Tomoyo Goto, Sunghun Cho, Tohru Sekino

**Affiliations:** The Institute of Scientific and Industrial Research, Osaka University, 8-1 Mihogaoka, Ibaraki, Osaka 567-0047, Japan; yjseo23@sanken.osaka-u.ac.jp (Y.S.); goto@sanken.osaka-u.ac.jp (T.G.); shcho@sanken.osaka-u.ac.jp (S.C.)

**Keywords:** low-temperature sintering, mineralization, bioactive glass nanoparticles, hydroxyapatite

## Abstract

Bioactive glasses are promising materials for various applications, such as bone grafts and implants. The development of sintering techniques for bioactive glasses is one of the most important ways to expand the application to biomaterials. In this paper, we demonstrate the low-temperature mineralization sintering process (LMSP) of glass nanoparticles and their crystallization behavior. LMSP is a novel process employed to densify glass nanoparticles at an extremely low temperature of 120 °C. For this new approach, the hydrothermal condition, mineralization, and the nanosize effect are integrated into LMSP. To induce mineralization in LMSP, bioactive glass nanoparticles (BGNPs, 55SiO_2_-40CaO-5P_2_O_5_, mol%), prepared by the sol-gel process, were mixed with a small amount of simulated body fluid (SBF) solution. As a result, 93% dense BGNPs were realized under a temperature of 120 °C and a uniaxial pressure of 300 MPa. Due to the effect of mineralization, crystalline hydroxyapatite (HAp) was clearly formed at the boundaries of BGNPs, filling particles and interstitials. As a result, the relative density was remarkably close to that of the BGNPs conventionally sintered at 1050 °C. Additionally, the Vickers hardness value of LMSP samples varied from 2.10 ± 0.12 GPa to 4.28 ± 0.11 GPa, and was higher than that of the BGNPs conventionally sintered at 850 °C (2.02 ± 0.11 GPa). These results suggest that, in addition to LMSP being an efficient densification method for obtaining bulk bioactive glasses at a significantly lower temperature level, this process has great potential for tissue engineering applications, such as scaffolds and implants.

## 1. Introduction

Ceramics and glass materials have been sintered to enhance their density and other properties, such as their mechanical properties, electrical and thermal conductivity, and so on. As is well-known, every stage of the sintering process, including mass transport, nucleation, and grain growth, requires a large amount of thermal energy [1]. In order to obtain a sintering density above 95%, the conventional solid-state sintering process for ceramic materials often requires a sintering temperature above 1000 °C [1,2]. Many sintering techniques, such as the application of high pressure and the addition of a liquid phase as an aid during sintering, have been investigated in a bid to reduce the sintering temperature required to fabricate ceramic and glass materials [3,4]. Moreover, more efficient sintering techniques, such as microwave sintering [5,6], spark plasma sintering [7,8], and flash sintering [9,10], which utilize electrical means, have been developed to densify ceramic materials at lower temperatures. Although these sintering processes have improved the sintering properties of ceramic materials, for example, producing a constant grain size and higher densification rate, the required sintering temperatures are still almost or more than 800 °C [11].

Contrary to the above sintering techniques, chemical reactions under hydrothermal conditions have also been employed to propel the sintering processes at significantly lower temperatures. For example, Gouda and Roy [12] carried out hot-pressing under hydrothermal conditions. They densified hydrated cement pastes with a small amount of water in a sealed vessel at a temperature of 250 °C and appropriate pressure levels that ranged from 340 to 520 MPa. Yamasaki et al. [13] prepared solidified amorphous and crystalline silica, calcium carbonate, zirconia, and barium titanate through hydrothermal hot-pressing (HHP) at 350 °C under 140 MPa. During HHP, an alkaline aqueous solution (NaOH) was used to promote mineralization under hydrothermal conditions. Recently, another sintering technique using hydrothermal conditions, called the cold sintering process (CSP), has been reported [14,15,16]. In CSP, a transient liquid phase containing constituent elements from inorganic particles in the presence of a small amount of an aqueous solution is utilized for mass transport. Then, the transient liquid phase is precipitated, leading to reprecipitation and densification of the inorganic particles at temperatures below 300 °C and under uniaxial pressures between 100 and 500 MPa. CSP has densified more than 50 species of inorganic materials, including various compounds ranging from binary to quaternary, such as oxides, bromides, chlorides, fluorides, phosphates, and carbonates [17]. However, the precipitate that promotes densification is basically the same as the parent inorganic materials used. Therefore, the densification in CSP is considered to be limited by the degree of dissolution of the component from the parent materials and the resulting degree of the transient state of the liquid.

Herein, we introduce a novel low-temperature mineralization sintering process (hereinafter abbreviated as LMSP). In a sense, it may be somewhat similar to previous processes, such as HHP and CSP. In terms of using moistened particles under a hydrothermal condition and a mold not completely closed to evaporate the water component, LMSP is similar to CSP. However, there are differences between the two processes. The densification mechanism of CSP involves a single solid phase where transport occurs by the pressure and concentration gradients at the particle interfaces under hydrothermal conditions [17]. As a result, densified inorganic material with a pure single phase can be obtained after CSP. On the other hand, through LMSP, biphasic inorganic materials can be obtained because LMSP is derived from mineralization using a supersaturated solution, which forms a certain mineral phase different from the inorganic base material. This formation and growth of the mineral at the interfaces of the base material particles can lead to their densification, forming biphasic inorganic materials.

In the present work, to demonstrate the mechanism of LMSP, ternary bioactive glass nanoparticles (BGNPs, SiO_2_-CaO-P_2_O_5_) as a starting material were prepared by the sol-gel process, and simulated body fluid (SBF) solution, which is a supersaturated solution, was used. Bioactive glasses have been widely investigated because of their peculiar characteristics, such as their biocompatibility and osteogenic capacity [18,19,20]. The dissolution of bioactive glass in contact with body fluids is critical for the mineralization process, forming a hydroxyapatite (HAp) layer, and the detailed mechanism of mineralization is described elsewhere [20,21]. The HAp layer tightly bonds with living bone tissue and allows the bioactive glasses to have high tissue integration and a high regeneration quality. In this sense, bioactive glasses have been increasingly studied for coatings of inactive metal materials for load bearing applications, and can obtain stable interfacial bonding with tissues and protect metallic surfaces from chemical degradation owing to body fluids [22,23,24]. There are two important reasons why we chose BGNPs for densification in LMSP. Firstly, as mentioned above, bioactive glasses exhibit a higher osteoconductivity, related to the formation of HAp, and the superficial HAp formation rate of bioactive glasses is therefore higher than that of bioactive ceramics [20]. Secondly, the surface area to volume ratio of nanoparticles is higher than that of micro- or sub-microparticles. Therefore, more reaction sites on surfaces of BGNPs enhance the nucleation during mineralization [25]. Overall, LMSP can be characterized as the new low-temperature densification technique that integrates the hydrothermal condition, mineralization, and nano-size effects. In this study, to apply LMSP for the fabrication of low-temperature sintered glass bodies, the densification of bioactive glass nanoparticles (BGNPs) through LMSP was carried out at 120 °C under a uniaxial pressure of 300 MPa using SBF solution that induced the mineralization. The formation and growth of the crystalline HAp played an important role in the densification of BGNPs. The effect of the mineralization on the densification behavior and the structural and mechanical properties of BGNPs sintered by LMSP were discussed. In addition, a comparison of LMSP and the conventional sintering process was made. To the best of our knowledge, this is the first study to densify BGNPs through mineralization under a hydrothermal condition.

## 2. Materials and Methods

### 2.1. Preparation of BGNPs Based on SiO_2_-CaO-P_2_O_5_

To obtain nanoscale SiO_2_-CaO-P_2_O_5_ ternary bioactive glasses (SiO_2_:CaO:P_2_O_5_ = 55:40:5 in mol%), a combination of the sol-gel process and the co-precipitation method, previously reported by Hong et al. [26], was employed. In brief, 7.639 g of calcium nitrate tetrahydrate and 9.167 g of tetra orthosilicate (FUJIFILM Wako Pure Chemical Corp., Osaka, Japan) were dissolved in deionized water (Arium^®^ pro, Sartorius, Göttingen, Germany)-ethanol (EtOH, 99.5%, FUJIFILM Wako Pure Chemical Corp., Osaka, Japan) solution (molar ratio of 2:1) at room temperature. The pH of the mixture solution was maintained between 1 and 2 using citric acid (FUJIFILM Wako Pure Chemical Corp., Osaka, Japan). After the solution became transparent, it was slowly dropped into 1.5 L of deionized water, including 1.078 g of ammonium dibasic phosphate (FUJIFILM Wako Pure Chemical Corp., Osaka, Japan), at a controlled pH of 11 by ammonia solution (FUJIFILM Wako Pure Chemical Corp., Osaka, Japan). The final mixture was strongly stirred for 48 h and subsequently aged for 24 h. The white precipitate was then obtained through centrifugation at 7000 rpm and washed three times with deionized water. The precipitate was freeze-dried (Freeze Dryer, FDU-2200, Tokyo Rikakikai Co., Ltd., Tokyo, Japan) with 2% polyethylene glycol (PEG, average Mn = 6000 g/mol, Sigma Aldrich Co. LLC, St. Louis, USA)-water solution to obtain spherical dense glass nanoparticles. The effect of the PEG chain on the size, morphology, and dispersity of the nanoparticles was reported by several previous studies [27,28]. Finally, the white BGNPs were obtained after calcination (Muffle furnace, EPDS-7.2K, ISUZU SEISAKUSHO, Co. Ltd., Niigata, Japan) at 700 °C for 3 h.

### 2.2. LMSP and Conventional Sintering of BGNPs

To readily apply LMSP to the obtained BGNPs, they were mixed with 45 wt% SBF solution synthesized by Kokubo’s method [29]. The BGNPs moistened by SBF solution were kept in a laboratory environment for 30 min to activate the surfaces of BGNPs for mineralization. The moistened BGNPs was then loaded into a 15 mm diameter cemented carbide cylindrical mold and heated at 120 °C under uniaxial pressing of 300 MPa using a silicon rubber heater (SAMICON SUPER340 II, Sakaguchi E. H VOC Corp., Tokyo, Japan) and a uniaxial press (Newton Press, NT-100H, NPa System Co., Ltd., Saitama, Japan). The heat treatment and mechanical pressing were performed for various sintering periods (0.5, 1, 2, 6, and 12 h). After the heating and pressing, the BGNP pellets were extracted from the mold and then washed with distilled water to remove excess ions that may have been left by the SBF solution. After washing, the pellets, which were approximately 1 mm thick, were placed in the oven and maintained at 120 °C overnight. The sintered samples obtained by LMSP are named LMSP0.5h, LMSP1h, LMSP2h, LMSP6h, and LMSP12h, depending on the sintering time.

To compare LMSP with the conventional sintering process, the green bodies, shaped by applying the same uniaxial pressure of 300 MPa, were sintered at 850 and 1050 °C for 2 h. The obtained conventionally sintered samples were named CS850 and CS1050, based on their sintering temperatures.

### 2.3. Characterization

The densities of the sintered BGNPs were measured through Archimedes’ method using absolute ethanol (EtOH, 99.5%, FUJIFILM Wako Pure Chemical Corp., Osaka, Japan) as a liquid medium. The relative densities were calculated by computing the ratio between the measured densities and theoretical densities of each sintered BGNP. The theoretical densities were obtained from the rule of mixture [30], based on the composition of each sintered sample from X-ray fluorescence (XRF, ZSX100e, RIGAKU Corp., Tokyo, Japan) analysis (see specific explanation in Appendix A). Microstructures of fracture surfaces of the sintered BGNPs were observed using a field-emission scanning electron microscope (FE-SEM, SU9000, Hitachi High-Tech Corp., Tokyo, Japan) under an acceleration voltage of 30 kV. Before observation, all of the samples were coated with Osmium by an Osmium plasma coater (OPC-60A, SPI Supplies, PA, USA). The structural change behavior was investigated by X-ray diffraction (XRD, D8 Advance, Bruker AXS GmbH, Karlsruhe, Germany). For XRD detection, CuKα radiation was generated at 40 kV and 40 mA, and the XRD patterns were obtained in the 2θ range from 10° to 60°, at a step size of 0.02. Grain boundary structures were observed using a transmission electron microscope with energy dispersed X-ray spectroscopy (TEM-EDX, JEM-ARM200F, JEOL Ltd., Tokyo, Japan). Samples for TEM and EDX mapping observation were prepared using a focused ion beam (FIB, FB2000, Hitachi High-Techn Corp., Tokyo, Japan). Chemical conversion of the sintered BGNPs was measured through Fourier transform infrared spectroscopy (FT-IR, FT/IR4100, JASCO, Tokyo, Japan). The compositional changes of LMSP samples depending on the sintering time were identified using XRF. The Vickers hardness test was performed using a Vickers hardness tester (FV-310 e, Future-tech Corp., Kanagawa, Japan). The Vickers hardness was measured six times for each sample under a 19.8 N applied load and 15 s holding time, and the average values were calculated.

## 3. Results and Discussion

### 3.1. Densification and Crystallization Behavior During LMSP

**Figure 1:**
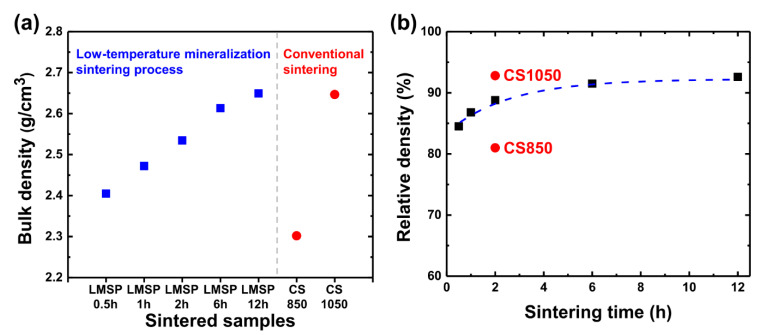
(**a**) Bulk density and (**b**) relative density against the sintering time of LMSP samples and cold sintering (CS) samples.

**Figure 2:**
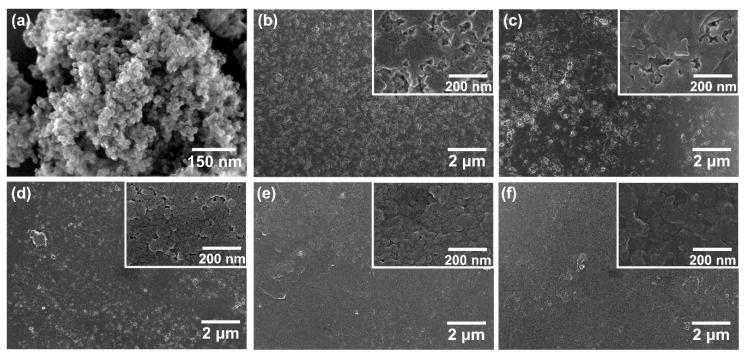
SEM images of (**a**) BGNPs, and BGNPs sintered by LMSP at 120 °C and 300 MPa for (**b**) 0.5, (**c**) 1, (**d**) 2, (**e**) 6, and (**f**) 12 h.

For the densification of BGNPs through LMSP, the ions, which would be precipitated between BGNPs, must be released from the glass networks. To promote this destruction in the glass networks, BGNPs that were mixed with SBF solution were stored for at least 30 min before pressing and heating. Figure 1 shows the bulk densities obtained from Archimedes’ method and relative densities against the sintering time of LMSP samples and CS samples. As shown in Figure 1a and Figure 1b, after sintering for 30 min, the bulk density and relative density of LMSP0.5h were determined to be 2.40 g/cm^3^ and 85% of the theoretical density (Table 1), respectively. Compared to BGNPs (Figure 2a), the neck formation between the BGNPs was observed in LMSP 0.5h (Figure 2b), which exhibits the typical microstructural feature of conventional sintering. As the sintering time increased, the pores, which can be found as spaces between particles on the surfaces, gradually decreased (Figure 2c–f). As a result, the bulk density and relative density of LMSP samples were increased up to 2.61 g/cm^3^ and 92% (LMSP6h) and 2.65 g/cm^3^ and 93% (LMSP12h), respectively. Compared to CS850, all of the LMSP samples exhibited a higher relative density (Figure 1b). For instance, the relative density of LMSP0.5h was higher than that of CS850 (81%). In addition, the relative density of LMSP12h was equivalent to that of CS1050 (93%) at a significantly lower sintering temperature.

According to the approximation curve of the relative densities (Figure 1b), the densification rate during LMSP dramatically increased for the first 0.5 h. From this result, we expected that the BGNPs would be rearranged according to the initial pressure at the beginning of LMSP, and the 85% relative density could then be achieved by chemo-mechanical dissolution and precipitation, as observed in CSP [14,15,16,17]. The existence of a small amount of liquid components from the SBF solution could also aid in the rearrangement and high compaction of the BGNPs. Moreover, the SBF solution could considerably enhance the precipitation process because the supersaturated solution could enhance the gradient of the ion concentration between the surface of BGNPs and the inside of the solution. After sintering for 6 h, densification appeared to be almost complete. It is expected that densification during the final stage of LMSP could be dominated by the formation and growth of calcium phosphate because the mineralization in the present work is also time-dependent. The formation of crystalline calcium phosphate such as HAp on the bioactive glasses takes several hours to several days of soaking in SBF solution [31,32].

**Figure 3:**
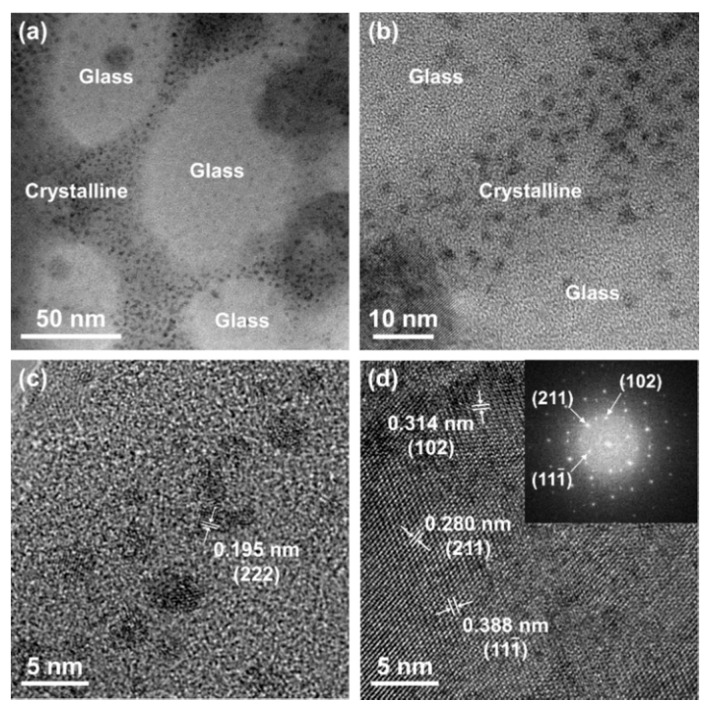
(**a**)**–**(**c**) TEM images from low magnification to high magnification and (**d**) high-resolution TEM image and corresponding electron diffraction pattern for a focused ion beam (FIB)-cut LMSP12h sample.

**Figure 4:**
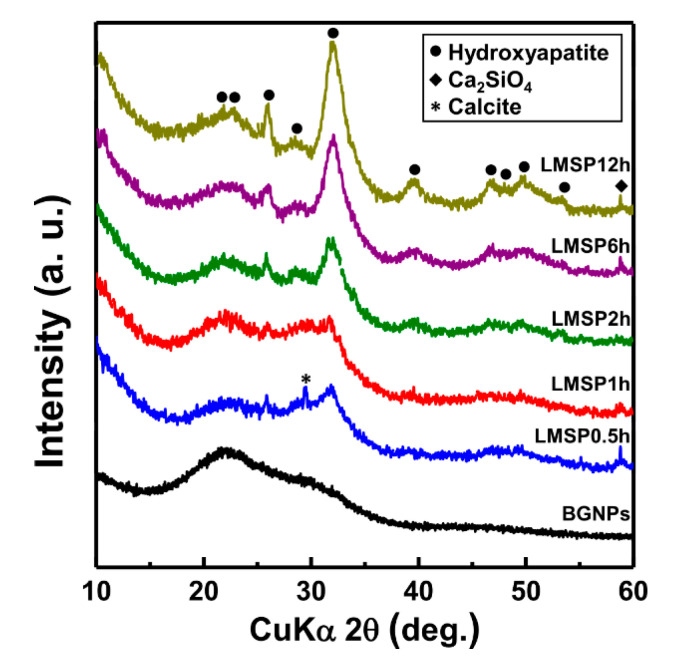
XRD patterns of BGNPs and LMSP samples in order of the sintering times from 0.5 to 12 h.

To show that mineralization affected the densification of BGNPs, TEM and XRD analyses were conducted. Figure 3 shows the cross sectional TEM images of LMSP12h, where the bright and dark parts refer to the BGNPs and the crystalline phase, respectively (Figure 3 a,b). As shown in Figure 3b, nanoscale crystalline phases were formed between the glass nanoparticles. The crystalline phase was approximately 2 to 4 nm and clearly identified as nano-sized hydroxyapatite by the selected-area electron diffraction patterns obtained through the TEM investigation (Figure 3d). These results indicate that BGNPs were surrounded by SBF solution and then densified by the formation of HAp through mineralization. According to the XRD patterns against the sintering time, in comparison with the XRD patterns of BGNPs, the crystalline peaks were first observed in LMSP0.5h (Figure 4). Then, after sintering for 2 to 12 h, the main peak of HAp (PDF no. 000-09-0432) was clearly separated at 2θ = 32.01° and assigned to the (211) diffraction peak. Most XRD peaks for LMSP12h also corresponded to those of HAp. Except for the crystalline HAp peaks, another crystalline peak at 2θ = 58.78° was also observed in several LMSP samples. This peak refers to a crystalline calcium silicate phase (Ca_2_SiO_4_, PDF no. 01-077-0420) that could be formed by the reaction between silanols and Ca^2+^ ions during mineralization [33]. In addition, a small peak at 2θ = 29.45° was only detected in the XRD pattern for LMSP0.5h, indicating the presence of calcite (PDF no. 01-072-1652), which could be formed by the presence of CO_2_ in the air during LMSP.

From these results, it is assumed that the crystalline HAp was formed within 6 h of LMSP. We suggest that this crystallization, accompanied by the densification, was affected by the following factors. First of all, the evaporation of the liquid component of SBF solution through the narrow space between the mold punch and die would enhance the supersaturation state of the phase at the interfaces of BGNPs, followed by the acceleration of the precipitation and mineralization of ions such as Ca^2+^ and PO_4_^3-^. According to the classical nucleation theory, the induction time (τ) for the formation of nuclei in solution can be described by the activation energy required for nucleation, $\Delta G^{*},$ as follows [34]:

(1) $$\tau =\frac{N^{*}}{J}=\frac{N^{*}}{\Omega }exp\left(\frac{\Delta G^{*}}{kT}\right)$$

(2) $$\Delta G^{*}=\frac{\beta \upsilon^{2}\gamma^{3}}{{\left(kT\ln S\right)}^{2}}$$

(3) $$\tau =Aexp\left(\frac{\beta \upsilon^{2}\gamma^{3}}{{\left(kT\right)}^{3}{\left(\ln S\right)}^{2}}\right)$$

where $N^{*}$ is the nucleation density, J is the nucleation rate, Ω is the pre-exponential factor, β is the shape factor, υ is the molecular volume, γ is the solid/liquid surface tension, k is the Boltzmann constant, T is the absolute temperature, and S is the supersaturation. From the final Equation (3), it can be explained that the induction time (τ), which is the time required to start the initial nucleation formation, is reduced by increasing the supersaturation (S). In addition, the particle size of BGNPs could also affect this rapid HAp formation because the larger surface area per volume of BGNPs compared to micro- and sub-microparticles provides more reaction sites, which can improve the rate of the nucleation of HAp [25]. Furthermore, the applied external pressure would enhance the dissolution of BGNPs and produce a chemical potential gradient near the particle surface that drives the nucleation of HAp. Therefore, the above-mentioned factors could have a significant influence on the formation of high-crystallinity nanoscale Hap, as well as the densification of BGNPs.

**Figure 5:**
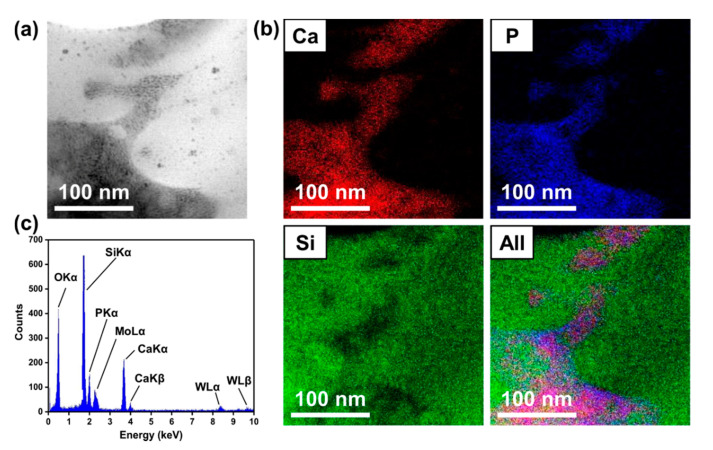
(**a**) STEM image, (**b**) EDX mapping images, and (**c**) elemental composition of LMSP12h.

Here, we also analyzed the distribution of HAp in the LMSP12h sample using TEM-EDX mapping analysis (Figure 5). As shown in Figure 5b, Ca and P, which are the main components of HAp, were dominant in the dark part. On the other hand, Si, related to BGNPs, was dominant in the bright part. It is therefore evident that the HAp was distributed in the boundaries of BGNPs, filling the pores and vacancies. Furthermore, no trace elements or impurities were observed in the sintered BGNPs according to the composition results (Figure 5c).

### 3.2. Chemical Conversion During LMSP

**Figure 6:**
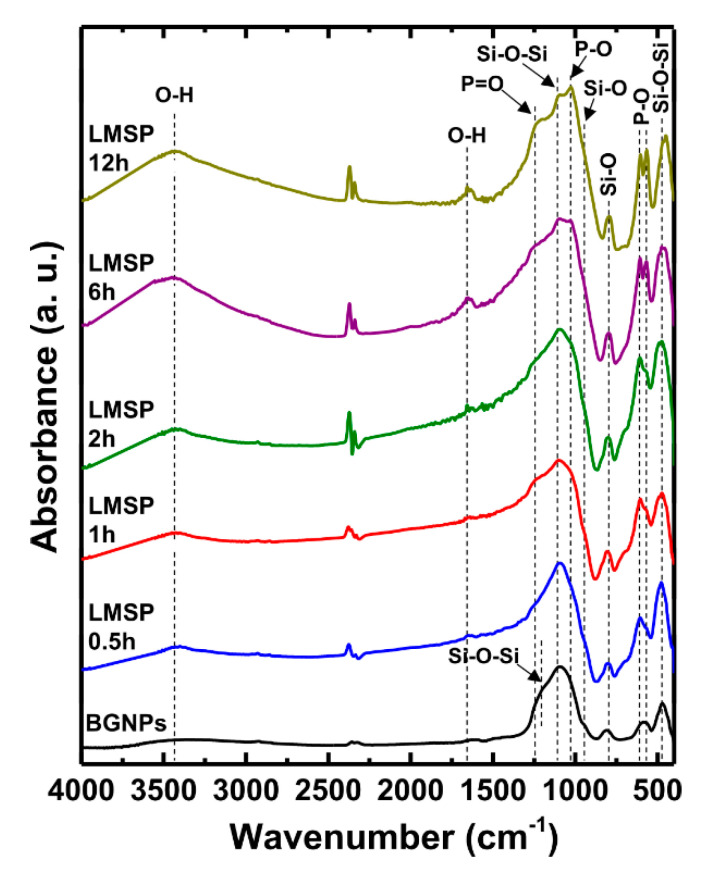
FT-IR spectra of BGNPs and LMSP samples in order of the sintering times from 0.5 to 12 h.

During densification, BGNPs can go through a chemical conversion through mineralization, so the structure conversion from BGNPs to LMSP12h was observed by FT-IR analysis (Figure 6). The FT-IR spectra of all samples showed typical absorption peaks of the silicate network in bioactive glasses; Si-O-Si bonds at 468 (symmetric bending), 1094 (symmetric stretching), and 1214 (longitudinal optical mode of asymmetric stretching) cm^−1^; and Si-O bonds at 794 (symmetric stretching of bridging oxygen) and 945 (stretching of non-bridging oxygen) cm^−1^ [35]. As the sintering time increased, additional absorption peaks, such as P-O bonds at 567 (asymmetric bending), 604 (asymmetric bending), and 1027 (symmetric vibration of PO_4_^3−^) cm^−1^ and the P=O group at 1252 cm^−1^, were newly observed [35,36]. In particular, two P-O bonds at 567 and 604 cm^−1^, observed after sintering for 6 h, coincide with the formation of crystalline HAp [33,37], and are consistent with the XRD patterns of LMSP6h and LMSP12h. Consequently, all of the newly observed peaks are related to the mineralization of BGNPs, and these results show that the mineralization of BGNPs can be successfully carried out using a small amount of SBF solution under a uniaxial pressure of 300 MPa and a temperature of 120 °C, unlike a normal SBF test condition [31].

### 3.3. Relationship Between Compositional Changes and the Densification Rate

**Figure 7:**
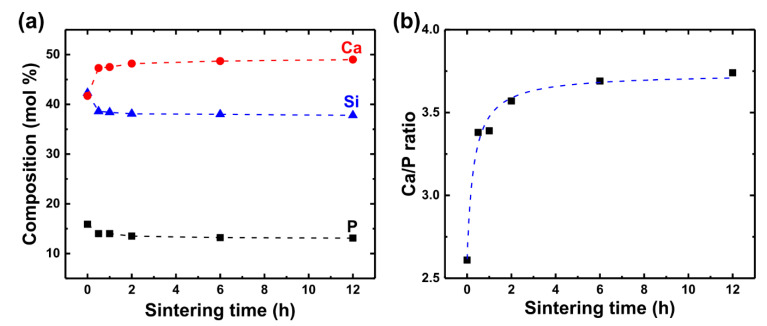
Changes in the (**a**) chemical composition and (**b**) Ca/P ratio of LMSP samples against the sintering time.

As mentioned earlier, during LMSP, the composition of sintered BGNPs is changed by the dissolution-precipitation and mineralization related to the densification of BGNPs. Therefore, to investigate the relationship between the compositional changes and densification, the compositions of each LMSP sample were compared against the sintering time through XRF analysis (Figure 7a). During the first 30 min, the composition of Si and P sharply decreased and finally declined by 4.5 mol% and 2.8 mol%, respectively. On the other hand, the composition of Ca dramatically increased during the first 30 min and showed a 7.3 mol% rise after sintering for 12 h.

Based on these compositional changes, the Ca/P ratio was also plotted against the sintering time (Figure 7b). From the results, it can be seen that the Ca/P ratio also rapidly increased during the first 30 min, and there were then almost no changes after sintering for 6 h, according to the approximation curve of Ca/P. These results were in good agreement with the results of the relative density because the increasing tendency of both parameters such as the Ca/P ratio and the relative density against the sintering time was very similar. Therefore, as with the approximation curve of the relative density, these results also indicate that the dissolution-precipitation process of LMSP mainly occurred during the first 30 min and was followed by mineralization. In addition, it can be concluded that the densification rate of BGNPs in LMSP depends on the mineralization rate of BGNPs.

### 3.4. Comparison of Vickers Hardness between LMSP and CS Samples

**Figure 8:**
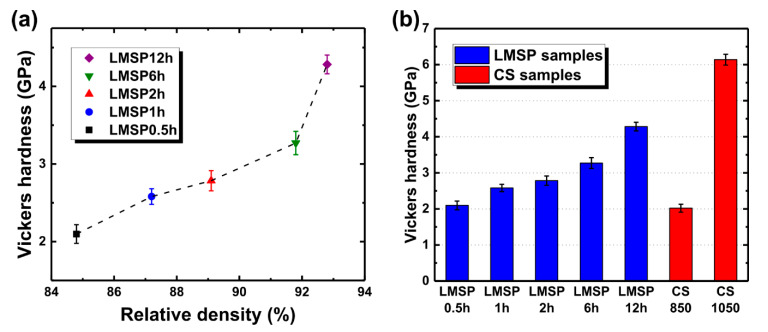
Vickers hardness of (**a**) LMSP samples against the relative density and (**b**) a comparison of the values for each fabrication condition of LMSP and CS samples.

The Vickers hardness test was performed to investigate whether BGNPs were merely densified through LMSP or BGNPs sintered through LMSP had sufficient mechanical properties applicable to their relative densities. The Vickers hardness of LMSP samples was described using the relative density (Figure 8a). Initially, the Vickers hardness of LMSP0.5h was 2.10 ± 0.12 GPa when its relative density was 85%. Then, as the relative density increased from 87% (LMSP1h) up to 92% (LMSP6h), the Vickers hardness also gradually increased from 2.58 ± 0.10 up to 3.27 ± 0.15 GPa. After this point, the Vickers hardness suddenly increased to 4.28 ± 0.11 GPa (LMSP12h), while the relative density difference between LMSP6h and LMSP12h was only 1%. This indicates that the growth of the crystalline HAp improved the hardness of LMSP12h because, as shown in Figure 6, the calcium phosphate phase was grown as crystalline HAp after sintering for 6 h, based on the observation of two P-O bonds at 567 and 604 cm^−1^. Moreover, according to the XRD patterns (Figure 4), the crystallinity of LMSP12h was the highest among LMSP samples.

**Figure 9:**
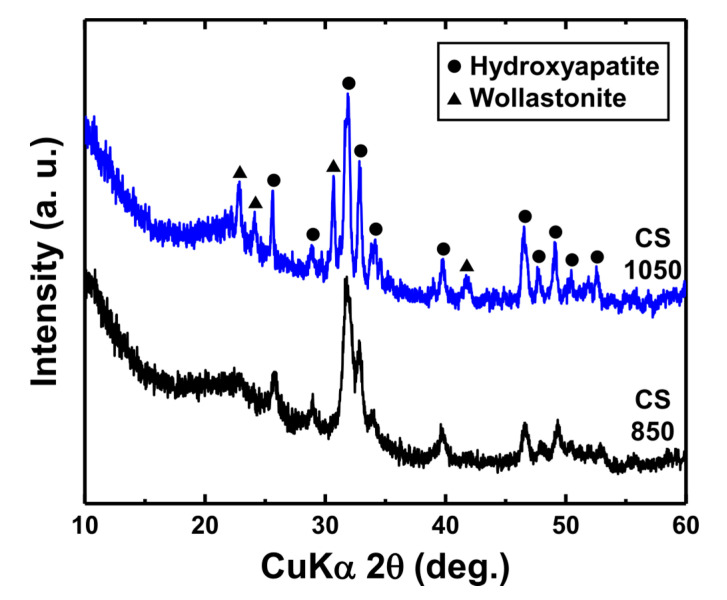
XRD patterns of CS850 and CS1050 sintered by the conventional process.

As mentioned in the previous section, we compared the Vickers hardness of LMSP samples with that of conventionally sintered samples (Figure 8b). The Vickers hardness of CS850 (2.02 ± 0.11 GPa) was lower than that of LMSP0.5h, which was consistent with the results of the relative density. As shown in Figure 9, CS850 contained crystalline HAp. Therefore, in this case, it was assumed that densification played a more important role in increasing the Vickers hardness compared to the crystallization of BGNPs. On the other hand, the Vickers hardness in CS1050 (6.14 ± 0.15 GPa) was almost 1.86 GPa higher than that of LMSP12h, even though their relative densities were almost similar. This can be attributed to the presence of wollastonite (PDF no. 00-043-1460) ceramic (Figure 9) in CS1050, in addition to that of HAp (PDF no. 00-009-0432). The mechanical properties, such as the fracture toughness, compressive strength, and bending strength of wollastonite crystals, have been reported to be superior to those of bioactive glasses and HAp [38,39,40]. Therefore, it can be concluded that the formation of wollastonite crystals contributed to the enhancement of the Vickers hardness of CS1050. However, in terms of the sintering temperature, LMSP (120 °C) was about seven and nine times lower than CS850 (850 °C) and CS1050 (1050 °C), respectively. At the same time, all of the LMSP samples showed better relative densities and Vickers hardness values than the conventionally sintered bioactive glass sample of CS850, and furthermore, the Vickers hardness of LMSP12h was 70% of that of CS1050. Consequently, although the calcination step operated at 700 °C was required in the present synthesis process of BGNPs, LMSP is an effective process for obtaining the sintering properties, equivalent to the conventional sintering process, at a significantly lower temperature in consideration of the densification process starting from the powder state.

## 4. Conclusions

LMSP was introduced for the novel low-temperature densification of bioactive glasses, and a relative density of 93% was achieved after uniaxial pressing at 120 °C for 12 h. During LMSP, the applied uniaxial pressure plays an important role in driving the densification, and the use of a supersaturated solution and hydrothermal conditions is required to accelerate the mineralization by significantly increasing the ion concentration gradient and supersaturation at the interfaces of BGNPs. Additionally, nano-sized particles would also enhance the nucleation rate of HAp during mineralization. Finally, the formation of the crystalline nano-HAp at the interfaces of BGNPs led to the final densification of BGNPs. The crystallization in LMSP greatly affects the increase in the relative density and Vickers hardness of the sintered BGNPs. At a sintering temperature of 120 °C, there is no viscous flow of BGNPs during LMSP, resulting in no dimensional shrinkage, except for a change in thickness by uniaxial pressing. Even though the Vickers hardness of CS1050 was higher than that of LMSP12h, from a comprehensive point of view, LMSP showed a similar or better relative density and Vickers hardness in comparison with the conventional sintering process, utilizing an extremely low sintering temperature. These results suggest that LMSP is an efficient and novel approach for obtaining bulk materials and could contribute to the low-temperature glass and glass-ceramics manufacturing process.

## Figures and Tables

**Table 1:** Composition of low-temperature mineralization sintering process (LMSP) samples and conventionally sintered bioactive glass nanoparticles (BGNPs) (wt%) and their theoretical densities (g/cm^3^) calculated by the mixture rule.

Samples	SiO_2_ (wt%)	CaO (wt%)	P_2_O_5_ (wt%)	Impurity (wt%)	Theoretical Density (g/cm^3^)
LMSP0.5h	45.8	37.3	16.8	0.1	2.846
LMSP1h	45.6	37.6	16.7	0.1	2.848
LMSP2h	45.2	38.3	16.4	0.1	2.854
LMSP6h	45.1	38.8	15.9	0.2	2.856
LMSP12h	45.1	39.0	15.8	0.1	2.861
CS850	45.4	38.2	16.1	0.3	2.849
CS1050	45.0	38.4	16.4	0.2	2.852

## Appendix A

In the present work, the theoretical density of each sintered sample was calculated from the chemical composition ratio of each sintered sample. By TEM, EDX mapping, XRD, and FT-IR analysis, it was found that the sintered samples were composed of bioactive glass nanoparticles (BGNPs) and nanoscale hydroxyapatite (HAp), similar to the structure of glass/ceramic composites. However, there is a limitation in assuming the concise volume fraction of BGNPs and Hap, as well as the theoretical density of sintered samples, because the HAp was formed by biomineralization (dissolution-precipitation) at the particle interfaces during LMSP. Additionally, the compositions of the sintered samples were changed by the formation of calcium phosphates through mineralization during LMSP. Therefore, we calculated the theoretical density using the rule of mixture based on the composition ratio of each sintered sample to discuss the densification behavior against the sintering time [30]. Additionally, the bulk density of each sintered sample obtained from Archimedes’ method was presented, accompanied by the relative density of the sintered samples (Figure 1).
